# Mountains as Evolutionary Arenas: Patterns, Emerging Approaches, Paradigm Shifts, and Their Implications for Plant Phylogeographic Research in the Tibeto-Himalayan Region

**DOI:** 10.3389/fpls.2019.00195

**Published:** 2019-03-18

**Authors:** Alexandra N. Muellner-Riehl

**Affiliations:** ^1^Department of Molecular Evolution and Plant Systematics & Herbarium (LZ), Leipzig University, Leipzig, Germany; ^2^German Centre for Integrative Biodiversity Research (iDiv) Halle-Jena-Leipzig, Leipzig, Germany

**Keywords:** flickering connectivity system (FCS), glaciation, Hengduan Mountains, mountain-geobiodiversity hypothesis (MGH), phylogeography, Pleistocene, Qinghai–Tibetan Plateau, refugia

## Abstract

Recently, the “mountain-geobiodiversity hypothesis” (MGH) was proposed as a key concept for explaining the high levels of biodiversity found in mountain systems of the Tibeto-Himalayan region (THR), which comprises the Qinghai–Tibetan Plateau, the Himalayas, and the biodiversity hotspot known as the “Mountains of Southwest China” (Hengduan Mountains region). In addition to the MGH, which covers the entire life span of a mountain system, a complementary concept, the so-called “flickering connectivity system” (FCS), was recently proposed for the period of the Quaternary. The FCS focuses on connectivity dynamics in alpine ecosystems caused by the drastic climatic changes during the past ca. 2.6 million years, emphasizing that range fragmentation and allopatric speciation are not the sole factors for accelerated evolution of species richness and endemism in mountains. I here provide a review of the current state of knowledge concerning geological uplift, Quaternary glaciation, and the main phylogeographic patterns (“contraction/recolonization,” “platform refugia/local expansion,” and “microrefugia”) of seed plant species in the THR. In addition, I make specific suggestions as to which factors future avenues of phylogeographic research should take into account based on the fundamentals presented by the MGH and FCS, and associated complementary paradigm shifts.

## Introduction

**FIGURE 1:**
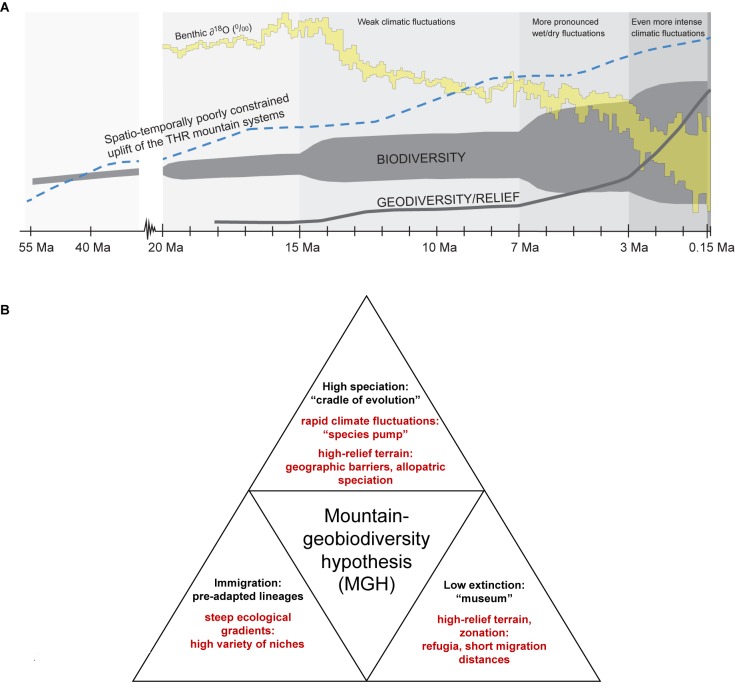
Two graphical summaries **(A,B)** referring to the “mountain-geobiodiversity hypothesis” by Mosbrugger et al. (2018). **(A,B)** Three boundary conditions as key for the origination of montane biodiversity hotspots: presence of lowland, montane and alpine zones (full altitudinal zonation); climatic fluctuations promoting a “species pump” affect; high-relief terrain with environmental gradients. **(A)** THR, Tibeto-Himalayan region. All temporally parallel trends are displayed schematically and without mutual vertical scaling. The oxygen isotope curve is based on Zachos et al. (2001, 2008), the uplift curve on Wang et al. (2011). The biodiversity evolution curve shows the merged data from Mosbrugger et al. (2018). The geodiversity curve is displayed according to the “mountain-geobiodiversity hypothesis” by Mosbrugger et al. (2018). Ma = million years ago. Reproduced from Mosbrugger et al. (2018) with modifications, with permission from the authors and the publisher (©John Wiley & Sons Ltd). **(B)** Three boundary conditions and their implications for montane biodiversity (see explanation in section “Introduction”).

The Tibeto-Himalayan region (THR) comprises the Qinghai–Tibetan Plateau (QTP), the Himalayas, and the biodiversity hotspot known as the “Mountains of Southwest China” (sometimes also referred to as “Hengduan Mountains biodiversity hotspot” in phylogeographic literature; Mosbrugger et al., 2018). Hotspots of biodiversity may often be found in areas that have undergone recent tectonic activity and harbor high topographic diversity (or ruggedness, sensu Körner et al., 2011). Yet, the origin and evolution of these hotspots remain relatively poorly understood. Until recently, for regions like the THR, most phylogenetic studies invoked orogenesis as the main driving force for the radiation of plants in mountains. The role of climatic fluctuations and key opportunities as well as key innovations as contributors to the establishment of high levels of mountain biodiversity was often neglected, though a few recent studies have employed statistical tests for more rigorously examining the impact of potential drivers of evolutionary plant radiations in the THR (e.g., Ebersbach et al., 2017a,b, 2018). More generally, several recent studies have indicated the importance of the interaction between traits and environments for generating high biodiversity levels in different taxa and ecosystems (e.g., interactions between traits and environments in cichlids, Wagner et al., 2012; for mountains and Ericaceae, Schwery et al., 2014; explored more generally, Bouchenak-Khelladi et al., 2015; Donoghue and Sanderson, 2015; for mountains and Saxifragaceae, Ebersbach et al., 2017a,b, 2018). The underlying causes for plant radiations in mountains are therefore likely to be multi-faceted, as has recently been shown for the Andes (Lagomarsino et al., 2016) and for the THR (Ebersbach et al., 2017b, 2018). Rather than geomorphological processes alone, the interaction of lineage-specific traits, complex geological settings and/or climatic modifications providing key opportunities are drivers of mountain biodiversity. Irrespective of the specific traits that taxa may have, the MGH (Mosbrugger et al., 2018) proposes that three boundary conditions are required to maximize the impact of mountain formation and surface uplift on regional biodiversity patterns and are key for the origination of montane biodiversity. These are (i) the presence of lowland, montane and alpine zones (full altitudinal zonation), (ii) climatic fluctuations for a “species pump” effect, and (iii) high-relief terrain with environmental gradients. Put differently, the MGH thus considers all three major factors responsible for high biodiversity in a given mountain region (Figure 1A). First, the presence of steep ecological gradients (full altitudinal zonation) would have allowed local/regional genera adapt to a high variety of niches along the elevational gradient and a range of differently pre-adapted lineages to immigrate into mountains. Second, rapid and profound Quaternary climate fluctuations would have fostered a “species pump” effect (here used to describe repeated cycles of connectivity and isolation as drivers of divergence; for original use of the term, with refugia acting as “species pumps” during climatic fluctuations, see Haffer, 1997), with highly rugged terrain providing geographic barriers for allopatric speciation. Third, highly rugged terrain, together with the presence of a full altitudinal zonation, would have provided refugia and short migration distances into favorable habitats during climatic changes (Figure 1B). In this way, mountains may be considered as “evolutionary arenas,” a term first used by Benzon (1996) in the context of cultural evolution, and recently coined by Peter Linder, Nicolei Nürk, and colleagues in an international symposium at the University of Bayreuth in 2018 as a concept for explaining the causes of accelerated evolution.

In addition to the MGH, which is setting the stage and covers the entire life span of a mountain, a complementary concept, the so-called “flickering connectivity system” (FCS), was recently proposed by Flantua and Hooghiemstra (2018) for the period of the Quaternary. As the new term already implies, this concept is putting a focus on connectivity dynamics resulting from repetitive climatic changes during the past ca. 2,6 million years before present, thus suggesting that fragmentation and allopatric speciation might not be the only factors for accelerated evolution of species richness and endemism brought about by climatic fluctuations and accompanying shifts of elevational belts. This is in line with the recently presented Mixing-Isolation-Mixing (MIM) model of speciation (He et al., 2018; compare comments by Abbott, 2018; Rieseberg, 2018). The MIM model supports that speciation may involve repeated cycles of mixing of genetically distinguishable populations (admixture), followed by geographic isolation until complete isolation is finally achieved. By permitting intermittent gene flow during speciation, MIM cycles can potentially generate species at higher rates than just by geographic isolation, which in turn means that speciation and the origination of high biodiversity levels in a region may be viewed in the same framework (He et al., 2018). Although the FCS is concentrating on the Pleistocene, Flantua and Hooghiemstra (2018, p. 172) emphasize that their concept builds on a “ghost from the past,” suggesting that historical connectivity left a strong imprint on present diversity of species. Areas that would have been more connected in the past would exhibit higher species richness today. In this way, Flantua and Hooghiemstra (2018) follow earlier studies which showed that mountain uplift was crucial for the evolution of landscapes and ecosystems, and that current biodiversity patterns are deeply rooted in the pre-Quaternary rather than being a mere product of the Pleistocene (e.g., see the review by Hoorn et al., 2010 for the Andean mountain system, or the review by Qiu et al., 2011 for the Sino-Japanese Floristic Region, including the QTP and Sino-Himalayan region). The FCS can be viewed as a valuable addition to the MGH for explaining montane biodiversity patterns, above and at species level as well as within species.

**FIGURE 2:**
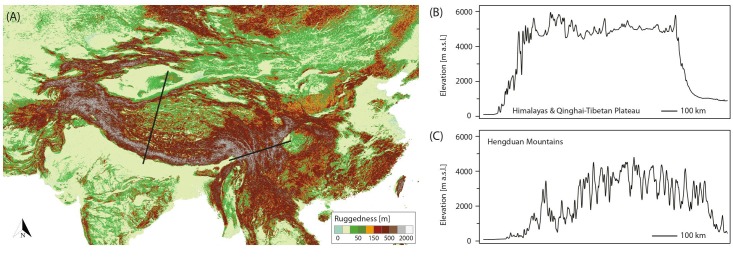
**(A)** Ruggedness map of the Tibeto-Himalayan region (THR), based on the definition by Körner et al. (2011). The topography of a mountain directly relates to the potential impact and frequency of connectivity breaks caused by Pleistocene climatic fluctuations, and thus the “species pump” effect as part of the mountain-geobiodiversity hypothesis (MGH), or more specifically, the expression of the flickering connectivity system (FCS). Left black line: location of transect through the Himalayas/QTP. Right black line: location of transect through the Hengduan Mountains. **(B,C)** Elevational transects through the Himalayas/QTP **(B)** and Hengduan Mountains **(C)** were derived from the Global Multi-resolution Terrain Elevation Data 2010 (GMTED2010) at 7.5 arc-seconds resolution.

**FIGURE 3:**
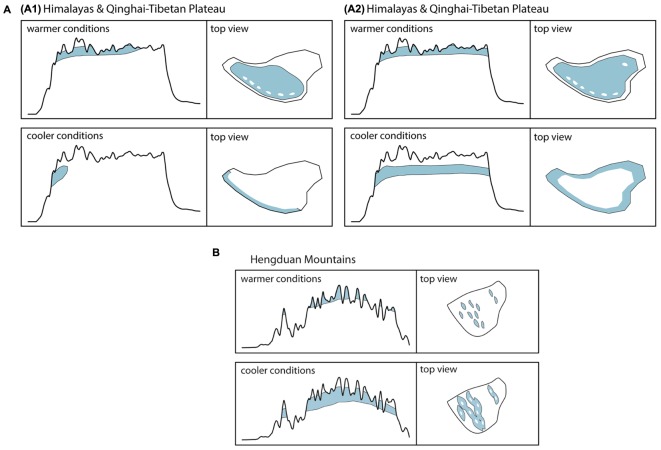
**(A,B)** Elevational shifts of high-mountain/alpine habitats (above tree line) of the THR during warmer and cooler periods as exemplified by the Pleistocene climatic fluctuations. Assuming temperature as the key factor for the distribution of habitats, cooler conditions will lead to a downslope shift associated with an increase in available habitat area and greater connectivity among populations in most mountainous regions of the Hengduan Mountains **(B)**. For lineages inhabiting high-elevation plateaus, like the QTP, however, this shift might result in a decrease of available habitat space, increased habitat fragmentation and a decrease in connectivity among populations **(A)**. Ecological adaptation of floristic elements will impact available habitat size and location: **(A1)** Reconstruction of habitat for high-mountain/alpine flora not adapted to very dry conditions. **(A2)** Reconstruction of habitat for high-mountain/alpine flora capable of inhabiting areas of very low precipitation.

Although originally introduced as a conceptual framework for the Pleistocene Andes (Flantua and Hooghiemstra, 2018) and later on used to quantify the temporal and spatial expression of habitat connectivity as biogeographical response to climatic fluctuations (Flantua et al., 2019), the FCS approach can as well be applied to the THR. Figure 2A shows the mountain area of the THR, based on ruggedness as defined by Körner et al. (2011), and Figure 2B,C show profiles of the Himalayas-QTP region and the Hengduan Mountains, respectively. The climatic fluctuations of the Pleistocene, driven by variations in the Earth’s orbit, would have caused substantial changes to plant distributions. Under the assumption that rates of evolutionary adaptation are slow relative to environmental rates of change, niche conservatism, and therefore tracking of suitable habitats, would have prevailed (Wiens, 2004; Pyron et al., 2015). Most plant species would therefore have followed temperature fluctuations by shifting their geographic range, with cold-adapted species and alpine ecosystems shifting downslope during glacial periods, and upslope during interglacials (Figure 3). Depending on mountain morphology, either cooler (glacials) or warmer (interglacials) conditions would have resulted in increased habitat size and thus often also connectivity for the alpine flora (compare Figure 3A vs. 3B). According to the FCS concept, diversification due to processes related to connectivity could have had a bigger impact on mountain biodiversity than merely isolation of populations (Flantua and Hooghiemstra, 2018). The four processes that are influenced by changing degrees of connectivity are: fragmentation, colonization (dispersal), intermixing, and hybridization (Flantua and Hooghiemstra, 2018). Finally, the “interaction between climate and topography,” which “defines when and where each process of the FCS occurs” could be expressed as a “unique identifier” of a mountain, the so-called “mountain fingerprint” (Flantua and Hooghiemstra, 2018). This mountain fingerprint stresses that eco-evolutionary processes occurred in a spatially and temporally diverse way in different mountain ranges, and that – importantly for phylogenetic and phylogeographic studies – discordant divergence times among multiple codistributed taxa are expected (compare section “Integration of Abiotic Factors into Phylogeographic Studies Conducted in the THR, and Related Paradigm Shifts”).

In contrast to plant phylogenetic studies above the species level, which so far have predominantly considered mountain uplift as a potentially important factor for plant evolution, plant phylogeographic studies at species level in the THR have traditionally equally considered the potential effect of Quaternary climatic fluctuations, mostly with regard to the impact of the Last Glacial Maximum (LGM), on population fragmentation (vicariance) and/or population expansion. Many of the phylogenetic as well as phylogeographic studies published to date also claimed to have found a potential impact of a very recent uplift of the QTP and associated recent establishment of the Asian monsoon system on plant evolution, a geological and climatic scenario that has recently been challenged (e.g., see detailed review by Favre et al., 2015; compare reviews by Renner, 2016; Spicer, 2017; radiometric dating and newly collected plant fossil archives by Su et al., 2018; fossil review by Deng et al., 2019). A solution for the various temporal scenarios of the “QTP uplift” cited in phylogeographic literature, which at first appear to be contradictory, is the differentiated consideration of geological histories of the different mountain systems located in the THR, as well as the different parts of the QTP itself. Put differently, it is important to (i) differentiate among the major mountain regions of the THR (i.e., QTP proper versus Himalayas versus Hengduan Mountains region), and (ii) between the major overall uplift of the QTP (up to ca. 3–4.5 km elevation in different parts during the Eocene to Miocene; old Paleogene proto-Tibetan upland not a vast plain, but more Andean-like shaped; Spicer, 2017; Su et al., 2018) versus younger local uplift above average plateau level (compare Figure 1 in Wang et al., 2011, for an earlier approximation of the elevation history of different parts of the QTP, or Wang C. et al., 2014, concerning older central versus younger marginal parts of the QTP; Favre et al., 2015; Spicer, 2017). Viewing the major mountain systems of the THR, the orogenetically younger and currently tectonically more active regions are located at the fringe of the QTP proper, in the Hengduan Mountains region and the Himalayas, and need to be considered separately from the QTP proper with regard to the potential general impact of mountain uplift on plant evolution (compare Xing and Ree, 2017). Favre et al. (2015) reviewed the geological literature of the THR for biologists in detail, providing a geological-climatic framework for evolutionary studies in the THR that has been widely used since then. Recent reviews (Favre et al., 2015; Renner, 2016; Spicer, 2017; Deng et al., 2019) support that major uplift of the QTP is dating back further in the past, and that some parts of the QTP may have been ca. 4 km high (similar to the present situation) already since the mid-Eocene, while a recent model-data comparison (Botsyun et al., 2019) suggests that the QTP may have reached elevations of more than 3 km only after the Eocene (see section “Geological Uplift History of the THR”), with no support for a general very recent “strong” uplift. Plant and animal fossil evidence supports the differentiated uplift history of the QTP and adjacent mountain systems (e.g., Spicer, 2017; Su et al., 2018; Deng et al., 2019). Whereas major consensus has been found for the geological and climatic history of the THR up until the Miocene/Pliocene (see section “Geological Uplift History of the THR”), the Pleistocene glaciation history of the THR is still a matter of ongoing debate (see section “Quaternary Glaciations in the THR”). At the same time, an increasing number of plant phylogeographic studies have found the late Pliocene/Pleistocene to be a key epoch for intraspecific diversification in the THR (see section “Main Phylogeographic Patterns of Seed Plant Species in the THR”).

About 90+ seed plant phylogeographic studies in the region of the THR have so far been published in internationally reviewed journals, on terrestrial as well as aquatic plant species, herbs as well as shrubs and trees, angiosperms as well as gymnosperms. A subset of these studies revealed strongly supported phylogeographic scenarios, allowing to take a closer look at emerging general patterns. Many phylogeographical studies discussed in this review suggested their results could be correlated to a recent “strong” uplift of the QTP, the latter which in the light of new evidence is not supported (see above, and in more detail in section “Geological Uplift History of the THR”). For this reason, here and elsewhere in this review, I will not refer to any *ad hoc* suggestion of a correlation of phylogeographic patterns to a young QTP uplift, but instead only report on the main outcomes of the phylogeographical studies based on supported facts. Based on the substantial advances of knowledge with regard to the geological and climatic/glaciation history of the THR in the past three decades, the amount of phylogeographic evidence accumulated for seed plants to date, and emerging paradigm shifts in the field, the specific aims of this review were to (1) summarize the geological uplift history of the THR and what is known about Quaternary glaciation in the THR, (2) summarize the main the phylogeographic patterns (“contraction/recolonization,” “platform refugia/local expansion,” and “microrefugia”) found so far in seed plant studies conducted in the THR, and (3) provide suggestions for future avenues of phylogeographic work in the THR. I thus hope to stimulate phylogeographic research in the area of the THR, one of the most biodiverse and geologically fascinating regions in the world.

## Geological and Glaciation History of the THR

### Geological Uplift History of the THR

Favre et al. (2015; compare Renner, 2016; Spicer, 2017) provided a detailed review of the geological and climatic (incl. monsoon) history of the QTP and Himalayas up until the Pleistocene for biologists. Mosbrugger et al. (2018) provided an update, considering additional literature, and proposed the MGH as a framework for future biological evolutionary investigations. Currently, there are no equivalent detailed reviews, written for a more general public of biologists, on the geological history of the THR for the Pleistocene. Concerning the QTP proper, this may be anyway regarded as only marginally relevant, as the main uplift basically predated the Quaternary (Favre et al., 2015; Renner, 2016; Spicer, 2017; Su et al., 2018; Deng et al., 2019). Even though research is far from establishing detailed regional surface elevation histories (see Wang et al., 2011 for an earlier approximation), there is increasing evidence that significant topographic features developed at least as early as the timing of the India–Eurasia collision (ca. 55–40 Ma, Eocene) (Yin and Harrison, 2000; Tapponnier et al., 2001; Rowley and Currie, 2006; Deng and Ding, 2016) and high Himalayan peaks developed no later than the early to mid-Miocene (Gébelin et al., 2013). The “Mountains of Southwest China,” the latter including the Hengduan Mountains, stand out from other mountain ranges in the THR due to several characteristics. In contrast to the QTP proper, parts of which may have reached 4,000 m elevation as early as 40 million years ago (Lippert et al., 2014; Renner, 2016; Spicer, 2017; Mosbrugger et al., 2018; Su et al., 2018; but compare Botsyun et al., 2019, who suggest only low to moderate, i.e., less than 3,000 m elevation during the Eocene), and the Himalayas, which were uplifted to significant elevations during the Miocene (Favre et al., 2015), the Hengduan Mountains, or at least parts thereof, are considered as relatively young (Miocene, late Pliocene) (Sun et al., 2011; Wang P. et al., 2014). These different geological histories have led to strongly contrasting evolutionary patterns throughout the region. While recent *in situ* diversification was found disproportionally more important for the species assembly in the Hengduan Mountains, Himalayan biodiversity was shown to have been largely influenced by immigration (Xing and Ree, 2017). Due to the different orientation of the THR mountain systems (Himalayas-QTP east–west, Hengduan north–south), one may also assume regional differences in extinction caused by past climatic variations, which could also have left their imprint on the observed diversification patterns as preserved in dated molecular phylogenetic trees (Xing and Ree, 2017). In addition, the Hengduan Mountains and the eastern Himalayas have been under the influence of the monsoon system (i.e., greater summer rainfall) ever since their orogenesis, whereas the QTP proper and the western Himalayas are almost entirely beyond the reach of the summer monsoon. Summing up, on phylogeographically relevant timescales, major uplift of the QTP proper would not have had an impact at the level of intraspecific divergence. Local uplift above average plateau level in the Quaternary (Wang et al., 2011) would hardly have impacted population diversification due to its relatively restricted geographic extent. The Himalayas and the Hengduan Mountains, though with a considerably younger history, nevertheless had reached significant elevation before the Pleistocene. One actual potential key factor of relevance for evolution at the level of species and populations in the THR, which has not yet received the deserved attention in either of the reviews by Favre et al. (2015), Renner (2016), or Mosbrugger et al. (2018), are the timing, locality and extent of Pleistocene glaciations.

### Quaternary Glaciations in the THR

The Himalayan-Tibetan orogen is the most glaciated region outside of the polar realms, with approximately 126,200 km^2^ glacier cover (Haeberli et al., 1989; Owen and Dortch, 2014). Glaciers are not only relevant to consider in terms of area not available for plant growth, but also in terms of their control on the development of topography, conditioning landscapes and focusing erosion, as well as governing tectonic feedbacks to the evolution of mountains (Brozovic et al., 1997; Zeitler et al., 2001; Norton et al., 2010; Willett, 2010), all of potential relevance for biotic evolution on mountains. Despite the importance of the THR, both in terms of glaciers being the source of numerous rivers providing freshwater to billions of people, and the orogen influencing regional and global atmospheric circulation, and its potentially useful geologic archive, Quaternary glaciation is not extremely well understood yet (Owen and Dortch, 2014). Apart from difficult accessibility to some parts of the region, challenges also lie in strong microclimatic variations within individual mountain ranges and valleys as well as significant variation of the two dominant climate systems (south Asian monsoon and mid-latitude westerlies) throughout the Quaternary, which would have resulted in asynchronous glaciation across the Himalayan-Tibetan orogen (Owen and Dortch, 2014). In addition to climate, topography has a strong influence on the nature of glacial systems throughout the orogen. Putting it short, the glacial system of the region is highly variable, which is true for both past and present (Owen and Dortch, 2014). Three main types of glaciers are distinguished in the THR: “(1) continental interior types in the central and western parts of the Tibet Plateau; (2) maritime monsoonal types in the Himalaya and in southeastern Tibet [and nearby Yunnan]; and (3) continental monsoonal types in eastern and northeastern Tibet” (Owen and Dortch, 2014, p. 17). Whereas glacier types (2) and (3) are usually highly active glaciers that have velocities up to several hundred meters per year (e.g., Seong et al., 2009), glaciers of type (1) would have much lower surface velocities, usually between 2 and 10 m per year (Owen and Benn, 2005; Owen and Dortch, 2014).

**FIGURE 4:**
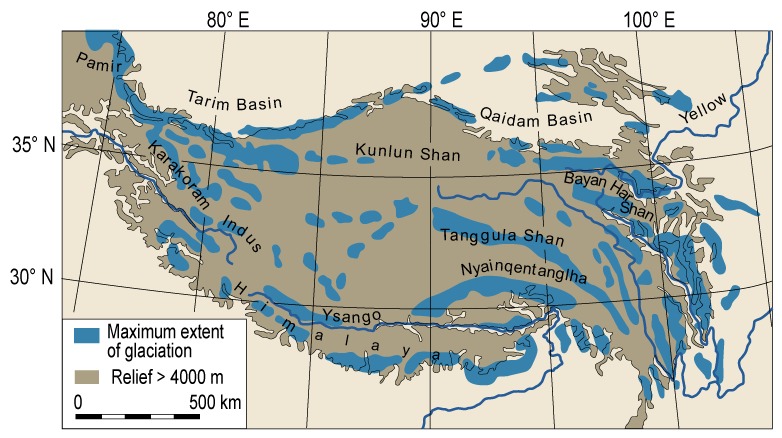
Selected reconstruction for the maximum extent of glaciation during the Last Glacial across Tibet and the bordering mountains of the Tibeto-Himalayan region (THR). Modified after Owen and Dortch (2014), with permission from the authors and the publisher (©Elsevier 2013, https://www.sciencedirect.com/science/article/abs/pii/S0277379113004599).

Several researchers of the twentieth and, increasingly, the 21st century have attempted to reconstruct the extent of Quaternary glaciation throughout the THR (historical overview summarized by Owen and Dortch, 2014; Supplementary Figure S1). Put shortly, in contrast to most studies, e.g., Kuhle (1998) argued for an extensive ice sheet covering most of the THR during the Last Glacial (ca. 115,000– ca. 11,700 years before present; Supplementary Figure S1). However, much evidence has been presented against the possibility that an extensive ice sheet could have existed across the QTP during the last 500 kyr; some controversies remain as to the apparently rather unlikely possibility for an ice sheet over the QTP during the early to mid Quaternary (ca. 2,6–1 million years ago; summarized and discussed by Owen and Dortch, 2014). Based on valley form alone, it may be regarded as rather unlikely that an ice sheet would have covered the Hengduan Mountains, as marks of glacial erosion (U-shaped valleys) are only limited to the upper catchment [e.g., transition from U-shaped (glacial) to V-shaped (fluvial) valley, close to the village of Yubeng, Meili Xueshan, Yunnan]. Figure 4 shows the most recent overall reconstruction of the maximum extent of glaciation during the Last Glacial across the QTP and the bordering mountains (modified after Owen and Dortch, 2014; compare also Yan et al., 2018). As outlined before, glacial extent in the THR would have varied considerably throughout the Quaternary, depending on climatic variations, moisture brought by monsoons, and the underlying topography. It is apparently highly debated whether glacial advances on the QTP occurred as a response to temperature cooling, or whether they were forced by an increase in moisture brought by the intensive Indian summer monsoon (Ou et al., 2014). In addition, concerning the influence of topography, it may be useful to consider the QTP rain shadow, with rain making it hardly past the Himalayas, leading to an arid climate on the leeward side of the mountain range, which could have influenced the amount of precipitation available for glaciation. For the monsoonal Hengduan Mountains it was suggested that glaciers would have been mainly triggered by changes in temperature (Ou et al., 2014), contrary to previous findings based on more limited sampling, which had proposed that during the LGM, despite cooler temperatures, the weakened south Asian monsoon and the associated reduced precipitation would not have been as favorable for glacier expansion as warmer times (Xu et al., 2010). The current view is in favor of Millennial time scale temperature fluctuations having possibly caused the multiple glacial advances in the Hengduan Mountains (Ou et al., 2014). For the western Himalaya, Hughes and Gibbard (2015) mentioned that glaciers would have reached their maximum extent well before the global LGM. The authors also claimed that until production rates and geomagnetic corrections have been better constrained for the THR, cosmogenic exposure ages may not be suitable to date the extension of glaciers at fine-resolution level (Hughes and Gibbard, 2015). Summing up, the complex interactions of temperature and precipitation fluctuations with glaciation at the background of topographical complexity in many parts of the THR (compare Figure 2B,C) will certainly pose a challenge for developing clearly defined hypotheses against which population genetic data can be rigorously tested.

Comparing the potential impact of Pleistocene glaciations on plant life among the three subregions considered in this review (QTP proper, Himalayas, and Hengduan Mountains), extinctions in the Hengduan Mountains region would likely have been relatively low due to the north–south orientation of its valleys, which could have allowed species to shift their distribution toward warmer southern ice-free areas (Xing and Ree, 2017). This scenario is supported by some studies showing long-term demographic stability of populations in this region (e.g., Fan et al., 2013; Liu et al., 2013). In contrast, the east–west orientation of valleys in the Himalayas–QTP region would likely have inhibited southward relocation, leading to greater population extinction, or at least contraction (Liu et al., 2013; Xing and Ree, 2017). In addition, the Hengduan Mountains are highly heterogeneous in terms of topography, featuring deeply dissected landscapes with steep elevational gradients (Figure 2C). In fact, the Hengduan Mountains are among the most rugged mountains of the world (compare Figure 2A) and such physiographic complexity seems to be a key factor promoting rapid radiations via allopatric speciation, as well as the evolution of narrow endemics (Hughes and Atchison, 2015; Hughes et al., 2015; Ebersbach et al., 2017a,b, 2018). Topographic complexity might also buffer extinction during climate change (e.g., Miocene cooling, Quaternary climate fluctuations) through the possibility of vertical displacement (Lancaster and Kay, 2013). Apart from the Hengduan Mountains, the Himalayas could also have profited from their greater ruggedness for promoting species diversity, in comparison to the large and less rugged areas of the QTP proper (Figure 2).

## Main Phylogeographic Patterns of Seed Plant Species in the THR

### Glacial Refugia and Postglacial Expansion in the THR: “Contraction/Recolonization,” “Platform Refugia/Local Expansion,” and “Microrefugia” as Three Main Phylogeographic Patterns

As outlined in the previous section, glacial climatic conditions would not inherently have been unfavorable or restrictive for plant survival due to a likely only patchy ice-sheet coverage of the THR (Figure 4). Some cold-tolerant species of different habitats and elevational belts would certainly have been able to survive in refugia in the THR throughout glacial periods. Examples (from higher to lower elevation) include *Rhodiola alsia* on alpine gravel slopes, *Aconitum gymnandrum* in alpine grasslands, *Potentilla glabra*/*P. fruticosa* in forests and alpine scrub, and *Juniperus tibetica* in forests (Gao et al., 2009; Wang L. et al., 2009; Wang L.Y. et al., 2009; Li C. et al., 2010; Opgenoorth et al., 2010). The situation in the THR can therefore be regarded as strikingly different from the one in (other) temperate zone mountain ranges more extensively covered by ice during the Pleistocene (e.g., the European Alps: Kropf et al., 2003; Christe et al., 2014, and references therein; Seguinot et al., 2018). For the following outline on main phylogeographic patterns in the THR, plant species with a current distribution on the QTP, some of them extending into the adjacent Himalayas and the Hengduan Mountains region, were considered.

Phylogeographic analyses in the THR have revealed three main evolutionary patterns for plant species (for a full list, see Supplementary Table S1), which, according to Gao et al. (2016) can be explained by the “contraction/recolonization” hypothesis, the “platform refugia/local expansion” hypothesis, and the “microrefugia” hypothesis (for a more general review of types of micro- and other mountain refugia, compare, e.g., Holderegger and Thiel-Egenter, 2009; Rull, 2009). Li et al. (2016) only differentiated two main refugial hypotheses, without naming them, but being comparable to the “contraction/recolonization” hypothesis and the “microrefugia” hypothesis as defined by Gao et al. (2016). Previously, Qiu et al. (2011; see their **Table 1**) had already presented a very useful compilation of plant phylogeographic research and listed patterns and major inferences, including proposed refugia, for the entire Sino-Japanese Floristic Region. Out of the 34 studies reviewed by Qiu et al. (2011), 14 dealt with species of the QTP and Hengduan Mountains region. Here, the authors identified two major refugial patterns for the latter regions, namely the “Re-colonization of the QTP from peripheral glacial refugia” (p. 231) and the “Glacial *in situ* survival on the QTP platform” (p. 232), thus being comparable to the “contraction/recolonization” hypothesis and the “platform refugia/local expansion” hypothesis as described by Gao et al. (2016). More recently, Zhang et al. (2018) differentiated a “nunatak” hypothesis from a “massif de refuge” hypothesis, where the first may be viewed as part of the “microrefugia” hypothesis, and the second as part of the “contraction/recolonization” hypothesis as defined by Gao et al. (2016). In the following, I will use the terminology used by Gao et al. (2016) when referring to the three concepts. It should be noted that while several authors have referred to various concepts as “hypotheses,” rarely they were strictly formulated as such. For the purpose of this review, I will mainly use the term “pattern,” as I will not test species phylogeographic histories against the three main “hypotheses,” but rather summarize the patterns found in published studies (see also Supplementary Table S1).

First, species following the “contraction/recolonization” pattern show a decline of genetic diversity from the edge to the center of the QTP proper. This would be caused by a retreat of QTP populations into refugia of lower elevation at the edge of the plateau during glaciations, followed by interglacial or postglacial recolonization of the plateau. This pattern has been observed in *Juniperus przewalskii* (Zhang et al., 2005), *Picea crassifolia* (Meng et al., 2007), *Metagentiana striata* (Chen et al., 2008), *Pedicularis longiflora* (Yang et al., 2008), and many other species in more recent studies (Supplementary Table S1). Second, the “platform refugia/local expansion” pattern describes high genetic diversity and a high number of private haplotypes present in populations clustered in a specific region on the plateau itself (compare “hotspots of high genetic diversity” on the QTP, Yu et al., 2018). This pattern would be caused by the presence of one or several isolated refugia on the plateau platform, with populations surviving in these local plateau refugia instead of retreating to edges at lower elevations during glaciations, followed by interglacial or postglacial range expansion. This pattern has been found in species such as *Potentilla glabra* (Wang L.Y. et al., 2009), *Aconitum gymnandrum* (Wang L. et al., 2009) or *Rhodiola alsia* (Gao et al., 2009, 2012), and several additional species in more recent studies (Supplementary Table S1). Third, species following the “microrefugia” pattern show a more or less even distribution of populations with high genetic diversity and frequency of private haplotypes across their current distribution range. This pattern would have been caused by the existence of multiple microrefugia during glacials throughout the current distribution range of those species in the THR. It was found in *Juniperus tibetica* agg. (Opgenoorth et al., 2010), *Hippophae tibetana* (Wang H. et al., 2010; Jia et al., 2011), *Rhodiola chrysanthemifolia* (Gao et al., 2016), *Potentilla fruticosa* (Li C. et al., 2010; Shimono et al., 2010; Sun et al., 2010), *Taxus wallichiana* (Gao et al., 2007), and many other species (Supplementary Table S1). There are also reported cases of population demographic stability or even expansion throughout the LGM (e.g., *Taxus wallichiana*, Liu et al., 2013).

In the following, I will address in more detail exemplary studies of phylogeographic research, and reference will be given to either of the three main patterns as described by Gao et al. (2016).

### The Hengduan Mountains as a Refugium

Du et al. (2017) investigated the phylogeographic history of *Quercus aquifolioides* (Fagaceae), an evergreen oak species occurring in the eastern parts of the THR (eastern QTP, Eastern Himalaya, Hengduan/Daxue Mountains, West Sichuan Plateau), by means of DNA data and fossil evidence. They suggested the species would have originated in the central parts of QTP, followed by subsequent dispersal eastwards toward the western Sichuan Plateau, and from there to the Hengduan Mountains region. The Hengduan Mountains biodiversity hotspot would have acted as a refugium in the late Neogene (before 2.8 Ma), with populations exhibiting great stability and low extinction in the Hengduan/Daxue Mountains, and haplotype radiation. Du et al. (2017) also found that the very rugged mountain ranges apparently would not have impeded extensive, presumably pollen-mediated, gene flow. The latter would have helped the populations of *Q. aquifolioides* to maintain a remarkably high genetic diversity throughout the entire region. For marginal parts of the Hengduan Mountains, and in particular the QTP proper, Du et al. (2017) suggested notable extinction-recolonization dynamics during the late Quaternary. The phylogeographic history of *Q. aquifolioides* is mainly in line with the “contraction/recolonization” hypothesis.

Li Y. et al. (2010) investigated several species of spruce, among them *Picea likiangensis*, *P. wilsonii*, and *P. purpurea* (Pinaceae), occurring in the southern and southeastern parts of the THR (Himalayas, Hengduan Mountains region, northeastern fringes of the QTP). They found high silent nucleotide diversity in these three species, all of which have a relatively restricted distribution. The authors reasoned that the observed genetic stability would suggest that the Quaternary climatic fluctuations would have had only little effect on the species’ distribution range. This is in contrast to European boreal species, many of which experienced severe bottlenecks during this period. Furthermore, Li Y. et al. (2010) stated that the high level of population differentiation for *P. wilsonii* and *P. likiangensis*, which occur at the eastern (*P. wilsonii*) and southeastern (*P. likiangensis*) fringes of the QTP, is also consistent with previous investigations on conifers from the same region, such as *P. asperata* (Wang et al., 2005), *P. likiangensis* (Peng et al., 2007), and *Pinus densata* (Ma et al., 2006). Presumably, the high population differentiation results from limited gene flow among populations due to the complex topography of their habitats, high mountains, and deep valleys (Wang et al., 2005; Peng et al., 2007). Additionally, the complex topography of the area might also have provided multiple isolated refugia during glacials (Tang and Shen, 1996; Peng et al., 2007). This could have reinforced the genetic population differentiation (Petit et al., 2003; Peng et al., 2007). In a more detailed follow-up study on *P. likiangensis*, Li et al. (2013) suggested that the species survived in multiple refugia throughout its range during the LGM and that the following postglacial expansions may have occurred mainly in limited parts along the distributional edges, where a single chlorotype or mitotype was fixed in the adjacent populations. The phylogeography of these *Picea* species shows highest resemblance to the “microrefugia” hypothesis.

In general, it seems to emerge that populations of plant species occurring in the Hengduan Mountains region tend to show long-term demographic stability (e.g., *Sophora davidii*, Fan et al., 2013; *Taxus wallichiana*, Liu et al., 2013). This seems reasonable, because the abiotic characteristics of this region can be viewed as more favorable for plants through time in comparison to either the QTP proper or the Himalayas (compare sections “Geological Uplift History of the THR” and “Quaternary Glaciations in the THR”). The exposure of the Hengduan Mountains to the moist and warm air of the Indian monsoon from the South would have provided a more stable environment at times of climatic fluctuations; greater latitudinal range due to north–south orientation of the mountains would have offered populations better possibilities to more easily escape unfavorable climatic conditions; and the highly rugged terrain (Figure 2C) could have provided more microclimatic refugia (see also Liu et al., 2013). Finally, it should be noted that the Hengduan Mountains region has also been suggested as a refugium for species with a restricted distribution there and in the Himalayas (e.g., Cun and Wang, 2010).

### The QTP as a Refugium

Lu et al. (2015) investigated the phylogeographic history of *Gentiana straminea* (Gentianaceae), a subalpine and alpine, rather cold-tolerant perennial with a widespread distribution in the eastern QTP region. Molecular dating estimates suggested that all haplotypes had differentiated before the LGM. Haplotype distributions based on both ptDNA and nrDNA data suggested expansions from the QTP to its outer edges. ENM suggested glacial survival of *G. straminea* on the QTP platform and continuous expansion to the platform edges. The findings implied that the species would have experienced initial diversification and glacial survival on the platform, and then continuously expanded its range to the QTP edges during the glacial period (Lu et al., 2015).

Investigation of the alpine aquatic herb *Ranunculus bungei* (syn. *Batrachium bungei*; Ranunculaceae) on the QTP, with observed inbreeding and clonal reproduction in the region (Wang Y.H. et al., 2010), revealed low within-population genetic diversity and high interpopulation genetic differentiation not coupled with a distinct phylogeographic structure (Wang Y.H. et al., 2010; Chen et al., 2014). Phylogenetic analyses revealed two main ptDNA haplotype lineages with divergence time dating back to the Neogene. Chen et al. (2014) suggested that *R. bungei* had survived the LGM and/or previous glacial periods on the QTP. Colonization or recolonization during the repeated range expansions would likely have replaced earlier haplotypes and a pre-existing genetic structure and would explain the non-significant phylogeographical structure (Chen et al., 2014).

Wang G.N. et al. (2014) suggested that the QTP endemic alpine herb *Pomatosace filicula* (Primulaceae) most likely survived the LGM at multiple unglaciated sites on the QTP, with most of the sites located above 4,000 m a.s.l. The authors’ phylogeographic analyses revealed a high level of differentiation among populations, but no significant phylogeographic structure, similar to the results of Chen et al. (2014).

Based on a phylogeographic study of the QTP alpine endemic *Aconitum gymnandrum* (Ranunculaceae), Wang L. et al. (2009) postulated the existence of four refugia for this species during the LGM, and that three of these refugia were located at high altitude on the QTP platform itself at that time. Coalescent simulation of chlorotype genealogies supported both an early Pleistocene origin of the two main clades found in *A. gymnandrum* and this ‘four-refugia’ hypothesis (Wang L. et al., 2009).

All phylogeographic examples given above are best in line with the “platform refugia/local expansion” hypothesis. Glacial *in situ* survival on the QTP platform was further supported for high-elevation junipers, *Juniperus tibetica* agg. (Cupressaceae; Opgenoorth et al., 2010), and *Potentilla fruticosa* (Rosaceae; Li C. et al., 2010; Shimono et al., 2010; Sun et al., 2010), both also occurring in regions surrounding the QTP proper, and showing highest resemblance to the “microrefugia” pattern (section “Plant Species With Multiple Refugia Across the THR”; Supplementary Table S1), as well as *Rhodiola alsia* (Crassulaceae; Gao et al., 2012), and *Potentilla glabra* (Rosaceae; Wang L.Y. et al., 2009), both best in line with the “platform refugia/local expansion” hypothesis (for *Rhodiola alsia*, Gao et al., 2012 also suggested an additional refugium in the Hengduan Mountains region).

### Plant Species With Multiple Refugia Across the THR

In a phylogeographic study on the alpine, herbaceous species *Saxifraga sinomontana* (Saxifragaceae), Li et al. (2018) found that only a few ancestral haplotypes/genotypes were widespread, while an extremely large number of them were restricted to single populations, which were scattered throughout the current distribution range of the species in the THR. Li et al. (2018) deducted the existence of microrefugia of this species during the Quaternary glaciations from these findings. Furthermore, the authors speculated that recent intraspecific radiation has occurred in the species, given the high haplotype richness, large proportion of private haplotypes, and shallow haplotype divergence found. Quaternary climatic fluctuations as well as large niche breadth may have triggered rapid intraspecific radiation in *S. sinomontana*. The phylogeographic history of *S. sinomontana* is mainly in line with the “microrefugia” hypothesis.

Gao et al. (2016) investigated the evolutionary history of a woodland- and shrubbery-inhabiting herbaceous species, *Rhodiola chrysanthemifolia* (Crassulaceae), occurring in southern Tibet, plus a few populations scattered across the interior of the QTP, and in the Hengduan Mountains region. They found high richness of private haplotypes as well as high total diversity versus relatively low average within-population diversity throughout the distribution range of the species. The phylogeographic history of *R. chrysanthemifolia* is mainly in line with the “‘microrefugia” hypothesis.

Gao et al. (2007) investigated the phylogeography of Chinese yew (*Taxus wallichiana*; Taxaceae), a tree species nowadays distributed over most of the southern and southeastern parts of the THR (Himalayas, “Mountains of Southwest China” biodiversity hotspot, down to the Sichuan basin), and beyond. They suggested a Sino-Japanese-Malesian origin of the species, with derived Sichuan basin and Sino-Himalayan haplotypes. Gao et al. (2007) found a westward Pleistocene migration event into the Hengduan Mountains, and deducted the existence of several Pleistocene refugia, such as in the East Himalaya, the Hengduan Mountains, around the Sichuan Basin, the East China region, the Tonkin Bay region, and Taiwan. As in the examples given above, this would be best viewed in line with the “microrefugia” hypothesis.

There are many more examples of plant species for which multiple refugia across the THR have been suggested (e.g., see *Juniperus tibetica* agg. and *Potentilla fruticosa* also listed also under section “The QTP as a Refugium,” and other examples given in Supplementary Table S1). The fact that many studies and plant species show a “microrefugia” pattern – and that many studies have found support for *in situ* glacial survival on the QTP platform (see section “The QTP as a Refugium,”) – provides support for the assumption that the THR has never suffered from extensive, geographically broad-scale glaciations (see discussion in section “Quaternary Glaciations in the THR”).

### Special Considerations for (Sub)Nival Plants

It should be noted that thus far most phylogeographic studies conducted in the THR have focused on plant species from forest or alpine grassland habitats (e.g., Li et al., 2011; Liu et al., 2013; studies in previous sections and in Supplementary Table S1). The flora of the subnival summits, e.g., of the Hengduan Mountains region, often exceeding 4,300 m a.s.l., has been largely neglected up to now. One of the few exceptions is, for example, a study by Luo et al. (2016), who found evolutionary histories and phylogeographical structures shared among four perennial herbs (*Eriophyton wallichii*, *Thalictrum squamiferum*, *Paraquilegia microphylla*, and *Chionocharis hookeri*), restricted to the subnival belt of the Hengduan Mountains region in China and adjacent countries. The four species harbored remarkably high levels of total genetic diversity for both plastid DNA and nuclear DNA markers, whereas values of intra-populational diversity were strikingly low (Luo et al., 2016). Both marker systems were strongly differentiated among populations but almost homogenous within populations. The authors suggested that such significant genetic structure and differentiation could mostly be due to drift-induced divergence associated with past demographic events during the Quaternary. Additionally, Luo et al. (2016) also found an almost simultaneous onset of diversification for the four study species during the mid-Pleistocene (ca. 0.73–0.47 Ma), which they suggested could be related to a period of severe glaciation. With respect to the more recent LGM, ENM suggested that the broad-scale distributions of all four species would have remained fairly stable over the last glacial/post-glacial cycle. Luo et al. (2016) attributed this to the fact that there never have been unified ice-sheets covering the entire QTP and adjacent mountains and that glacier expansion was less pronounced than in other parts of the Northern Hemisphere (see section “Quaternary Glaciations in the THR” and Figure 4).

## Conclusion

### Suggestions for Future Avenues of Phylogeographic Work in the THR: Emerging Approaches and Paradigm Shifts

#### Consideration of Species-Specific Traits to Phylogeographic Patterns in the THR, and a Related Paradigm Shift in Comparative Phylogeography

More studies are needed that focus on estimating and understanding the differences in population biology, life cycle, habitat ecology, or dispersion ability of montane plant species of the THR. Having a representative number of studies for plant species adapted to different elevational zones will allow, for example, comparing their Quaternary evolutionary histories, potentially enabling to find correlations between traits and either of the three established main phylogeographic patterns (“contraction/recolonization,” “platform refugia/local expansion,” or the “microrefugia” hypothesis). Different patterns of glacial isolation and postglacial range expansion across the same area are expected for different species (compare e.g., Taberlet et al., 1998; Hewitt, 1999; Kropf et al., 2003, 2006 for European mountains). Phylogeographic structures of different plant species occurring in the THR, with different ecologies and/or altitudinal preferences, will have been shaped by asynchronous range change cycles during glacials and interglacials, as has been shown for European mountains (Kropf et al., 2003). The importance of understanding “species’ characteristics (that is, ecological traits and biotic interactions) because these influence species coexistence, persistence and extinction, affect responses of species to environmental gradients, and determine their functional role in ecosystems” was very recently also highlighted in a global review of climatic and geological influences on mountain biodiversity (Antonelli et al., 2018, p. 722).

The importance of trait-based hypotheses in phylogeographic research, and the necessity for a paradigm shift, was recently emphasized by Papadopoulou and Knowles (2016, p. 8018) who illustrated “how refined hypotheses based on taxon-specific traits can provide improved predictive frameworks to forecast species responses to climatic change or biogeographic barriers while gaining unique insights about the taxa themselves and their interactions with their environment.” Papadopoulou and Knowles (2016) made the point that, so far, classical comparative phylogeographic work has concentrated on genetic surveys trying to reveal concordant phylogeographic patterns/discontinuities, addressing questions like, e.g., the inference of Quaternary refugia. They argued that one of the limitations coming with such an approach, relying on the phylogeographic concordance criterion, was the “tendency to disregard discordance as uninteresting and attribute lineage-specific patterns to stochastic effects” (p. 8019), as they would not be fitting the expected main patterns. In addition, the concordance criterion would naturally have favored the investigation of abiotic/extrinsic factors (e.g., geological and associated climatic/glaciation events), at temporal scales which would have affected codistributed taxa similarly, over the investigation of biotic/intrinsic factors (e.g., ecological or life history traits; Papadopoulou and Knowles, 2016). The same authors claimed that this would have contributed to an “imbalance in the perceived relative importance of extrinsic factors in structuring genetic variation (as opposed to the influence of intrinsic factors)” (p. 8019). As an example, Papadopoulou and Knowles (2016) mention that when evaluating the effect of Pleistocene glaciations on the evolutionary history of co-distributed montane plant taxa, spatially discordant phylogeographic patterns would often be deemed inconclusive, whereas they could provide insights into the interaction between species’ ecology and climatic change. A way out of this dilemma is bridging the gap between pattern (phylogeographic signal) and mechanism (traits) by a shared framework, considering the relative influence of both extrinsic and intrinsic factors in structuring genetic information (Papadopoulou and Knowles, 2016). New comparative phylogenetic methods will have to gain insights from discordant patterns rather than only quantifying congruence. Rather than applying species-specific or trait-based explanations in an *ad hoc* manner (i.e., after discordance is observed), an appropriate statistical model-based framework would accommodate and interpret phylogeographic discordance. As one example of such an approach, Papadopoulou and Knowles (2016) present a recently developed methodology integrating distributional, demographic and coalescent modeling (iDDC; He et al., 2013) with approximate Bayesian computation (ABC; Beaumont et al., 2002). This methodology provides “a framework for evaluating the relative probability of alternative hypotheses based on species-specific expectations under a diverse array of models that can accommodate differences in population dynamics over space (e.g., habitat heterogeneity) or time (e.g., shifting climatic conditions), but also differences in taxon-specific demographic processes because of disparate natural histories or ecologies” (Papadopoulou and Knowles, 2016, p. 8021). What remains challenging for the researcher, of course, is to feed this approach with meaningful data and hypotheses. Good knowledge of the biology and traits of the species under investigation, and the most relevant abiotic factors will be key ingredients to arrive at meaningful conclusions. Even if the focus of phylogeography in the THR may stay on inferences about historical/abiotic factors, a shift toward trait-based considerations in comparative phylogeography of this region may become more broadly relevant in the future. Especially for (sub)nival vegetation, which constitutes a highly fragmented plant cover above the belt of closed alpine vegetation, and mainly comprises cold-resistant, xerophilous, long-lived and/or slow-growing species (Wu et al., 1987; Li and Li, 1993; Körner, 2003; Xu et al., 2014; Luo et al., 2016), low biotic complexity results in abiotic factors, particularly climate, dominating over biotic factors, such as competition (Pauli et al., 2003; Luo et al., 2016). The consideration and implementation of biotic factors into methodological approaches will therefore be more relevant for plants of lower vegetational zones (alpine and below) in the THR.

Last, it should be noted that phylogeographic studies in the THR have so far concentrated on the investigation of endemic taxa (although there are some exceptions, e.g., Luo et al., 2016). For more general insights into the colonization history of the THR and the study of dispersal pathways to, within, and from the THR, more species with a broader distribution need to be studied in the future.

#### Integration of Abiotic Factors Into Phylogeographic Studies Conducted in the THR, and Related Paradigm Shifts

Starting in deep time, the MGH (Mosbrugger et al., 2018) provides a conceptual framework for the entire life span of the THR, including the geological evolution of the mountains (incl. geological heterogeneity), the evolution of the modern monsoon system(s), as well as the intensification of climatic fluctuations toward the present (Figure 1), and the consecutive build-up of biodiversity during the process of mountain formation. Due to some uncertainties in the exact timing of events in deep time (many million years ago), the MGH provides a coarse backbone for the evolutionary “ghost from the past.” Building upon this “ghost from the past,” the FCS approach (Flantua and Hooghiemstra, 2018) provides a valuable and testable framework for the Pleistocene, for which population-level genetic data are informative. As outlined in the introduction, the FCS focuses on connectivity dynamics of mountain systems brought about by elevational shifts triggered by climatic fluctuations. The novelty of the FCS approach lies in quantifying both the frequency and the duration of population connectivity based on GIS models that reconstruct vegetation distribution through time using exceptionally long fossil pollen data (Flantua et al., 2019). The paradigm shift here therefore lies in the explicit consideration of both frequency and duration of climatic changes over a longer time span, resulting in quantifiable population connectivity, rather than discussing or attributing single extreme climatic events, like the LGM (not representing a typical scenario during the Pleistocene climatic fluctuations), to observed phylogeographic patterns in an *ad hoc* manner. Population genetic data derived from high-throughput sequencing approaches, allowing for a finer-scale temporal resolution of and confidence in molecular dating, will certainly allow a more precise investigation of potential correlations between abiotic changes and biotic evolution at the level of populations and species (see, e.g., Ren et al., 2018, who found much older age estimates for *Primula* species in the THR based on RADseq data than previous estimates had yielded based on a few plastid markers). They also have the potential to rigorously test the postulated “species pump” effect, and MIM model of speciation, respectively (see section “Introduction”) for the THR, and provide further insights into the start and maintenance of interspecific divergence in the context of changing environments in mountain systems of the THR (e.g., Ren et al., 2018), gene flow between species or intraspecific lineages as cause of hybrid speciation, introgression or homogenization of certain genomic regions (e.g., compare Li Y. et al., 2010, Li et al., 2012; Hu et al., 2016), and many other related population genetic questions (Liu et al., 2012).

Uncertainties concerning the impact of climatic fluctuations on monsoonal precipitation patterns and the extent of glaciation exist for most parts of the THR (e.g., Ou et al., 2014; Owen and Dortch, 2014; Hughes and Gibbard, 2015; Yan et al., 2018). The impact of climatic fluctuations and precipitation patterns on species’ distributions through time are expected to vary greatly depending on topography of each respective region of the THR (Figure 2), or put more precisely, depend on the “mountain fingerprint,” as defined by Flantua and Hooghiemstra (2018; see section “Introduction”). The “mountain fingerprint” is considered to be a unique identifier, as mountains have different topographic profiles and different mountain regions experienced different degrees of habitat connectivity as a result of climatic fluctuations (as the same authors show in the Northern Andes; Flantua et al., 2019; compare Figure 3 for the THR). The authors postulate that this “mountain fingerprint” defines in a spatially and temporally complex manner where and when each of the four processes inherent to the FCS [fragmentation, colonization (dispersal), intermixing and hybridization] occurred, and argue that these processes together define the legacy effect of historical connectivity in contemporary species richness and endemism.

The introduced concept (Flantua and Hooghiemstra, 2018) and quantification of the FCS in mountain regions (Flantua et al., 2019) acknowledge the inherent complexity of mountain landscapes through time, and that observed phylogeographic patterns are bound to inherit this complexity. Considering the reasoning of Papadopoulou and Knowles (2016; section “Consideration of Species-Specific Traits to Phylogeographic Patterns in the THR, and a Related Paradigm Shift in Comparative Phylogeography”) about the contribution of species-specific traits to phylogeographic patterns and the need to welcome discordant patterns, the FCS and “mountain fingerprint” should be viewed as a desirable integrative part of future methodological approaches, which then combine both biotic, trait-based information, and abiotic, mountain-based information. Input for such future phylogeographic approaches, which would then not only be applicable to the THR, but mountains worldwide, will be required from multiple disciplines. Notwithstanding, abiotically as well as biologically well-informed hypotheses should always constitute the cornerstones of future phylogeographic work in the THR, and elsewhere.

**FIGURE 5:**
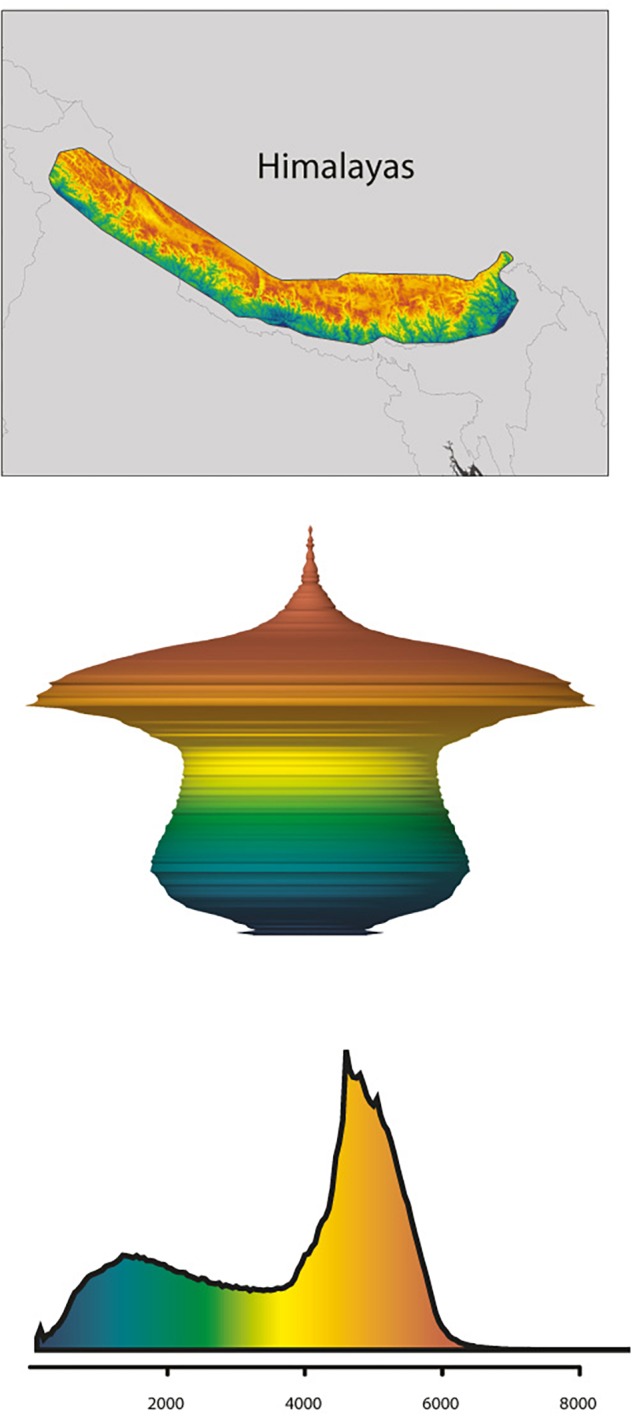
Example of the Himalayas following the hourglass hypsographic classification. **(Top)** Shuttle Radar Topography Mission topography (SRTM) for the Himalayas (an hourglass range). **(Middle)** Three-dimensional model spindle representing the relative surface area available (*xy*-planes) with elevation (*z*-plane), derived from the Digital Elevation Model (DEM). **(Bottom)** Hypsographic curve derived from the DEM, with elevation in m a.s.l. along *x*-axis. Colors relative to a maximum elevation of 8,685 m observed in the Himalayas. Adapted by permission from the authors and Springer Nature Customer Service Centre GmbH, Nature Climate Change, Global mountain topography and the fate of montane species under climate change (Elsen and Tingley, 2015; © 2015 Macmillan Publishers Ltd.).

In line with the fundamentals presented by the MGH and FCS (incl. the consideration of the “mountain fingerprint”), mountain morphology may be a key factor for explaining phylogeographic patterns rooted in the Pleistocene, but also predict future scenarios. More specifically, Elsen and Tingley (2015) differentiated four mountain hypsographic classifications, which would have an impact on surface area distribution of different elevational zones available for plant growth. For example, in contrast to the Rocky Mountains (“Diamond” type) and the Alps (“Pyramid” type), the Kunlun Mountains (“Inverse pyramid” type) and the Himalayas (“Hourglass” type) are both characterized by an area increase with elevation, which has important implications for the fate of montane species under climate change. Specifically, some species responding to climate warming by shifting upslope may actually benefit through increases in available area (Figure 5; Elsen and Tingley, 2015; compare Figure 3A). Given that the mountain systems of the THR had reached elevations comparable to present-day when Pleistocene climatic fluctuations would have acted upon biota, this in turn means that plant species adapted to different elevational zones in the THR would have been differently affected (either positively, or negatively) by glacial and interglacial periods (Figure 3). Summing up, the long-held, European-biased (e.g., Alps) general view and inherent assumption that mountain surface area will generally decrease with increase in elevation, does not apply for all mountain systems of the THR (Figure 3). This insight and the consequences it brings for phylogeographic hypothesis-testing may therefore be regarded as another paradigm shift which will need to be considered in future studies.

**FIGURE 6:**
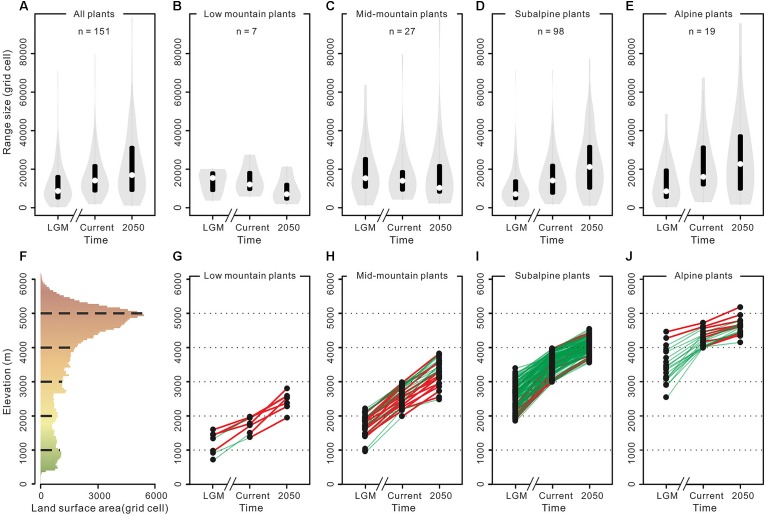
Trends in variation in the range size and altitude of a representative sampling of plants occurring in the Tibeto-Himalayan region (THR), in response to climate warming, by Liang et al. (2018). **(A)** Range size change trend for all plants. **(B–E)** Range size change trends for plants in different elevation zones. **(F)** The surface area available along the elevation in the THR, derived from the Digital Elevation Model (DEM). **(G–J)** Range size change trends for each plant species in different elevation zones. The white plotted points in **(A–E)** represent means of plants range size. The black plotted points in **(G–J)** represent the elevation of the centroid for each plant. A green line indicates that the range size is expanding. A red line indicates that the range size is contracting. Reproduced with minor modifications from Liang et al. (2018) with permission from the publisher (©2018 John Wiley & Sons, Ltd).

As a practical example of the impact of mountain morphology on the effect of past and future climate change on species elevational shifts and range size change trends in the THR, Liang et al. (2018) modeled the response of 151 representative seed plant species (“ecologically dominant species, structural species among the vegetation or species that are endemic but have wide ranges”; belonging to 87 genera of 41 families) occurring in subalpine and alpine belts to gradual warming in the THR, from the LGM to 2050 (Figure 6). As expected, all plant species showed a general upward trend, moving toward higher elevations (Figure 6G–J). Contrary to the previous long-held expectations as outlined further above, the authors found that most of the investigated subalpine and alpine plant species would even profit from an increase in range size in the course of their elevational upward shift (Figure 6A–E). Without information on mountain morphology, this might, at first sight, seem surprising given that land surface area was generally expected to decrease with increasing elevation in mountains (see above). However, because of the heterogeneous topography of the THR, and the large size of the QTP proper, land surface area is peaking at higher elevations (Figure 6F; compare Figure 5 for the Himalayas only; Elsen and Tingley, 2015). This is a good illustration of how plant species will be affected differently in different mountain systems by climatic fluctuations, depending on each mountain’s morphology.

#### Additional Considerations for Mountain Comparisons

What has not yet received the desired attention in either the MGH or the FCS is latitude of the mountain (but see Liang et al., 2018; and earlier comments by Qiu et al., 2011). Certainly, not only W–E or N–S direction of a mountain with respect to moisture fluxes (Antonelli et al., 2018), but also the latitudinal position on the Earth’s surface could be a key factor for the severity of the impact of climatic fluctuations on the mountain’s biota. Qiu et al. (2011, p. 240) mentioned that, unlike the biota of colder latitudes, the montane flora of China did not follow “a basic expansion-contraction (EC) model of latitudinal range change.” The authors claimed that studies rather indicated survival and divergence of populations through many glacial cycles in warmer temperate or (sub-)tropical regions of Asia, resulting in greater phylogeographic subdivision than those at colder latitudes.

### Final Notes

In the past, a considerable number of biological (e.g., phylogenetic and phylogeographic) as well as glaciation studies has relied on a too young “strong” uplift scenario for the QTP, which could have hampered at least some progress regarding the advancement of our understanding of the correlation between phylogeographic patterns and abiotic conditions. Future studies should take into account the latest advances in knowledge, based on different methodological approaches and newly collected fossil evidence (see section “Introduction” and section “Geological Uplift History of the THR”).

What is still badly needed is a summary of the current views on the Pleistocene glaciation and climatic history of the THR for biologists, as a geological-climatic framework against which clearly defined phylogeographic hypotheses may be tested rigorously, instead of applying explanations that fit the observed results *ad hoc* (i.e., after analysis of the data), as has been done in the past. Recently or newly developed dating techniques in glacier research that include optically stimulated luminescence and terrestrial cosmogenic nuclide surface exposure dating should hopefully allow glacial successions throughout the QTP and the bordering mountains to be dated and correlated somewhen in the near future (Owen and Dortch, 2014; Saha et al., 2018). Likewise, plant phylogeographers are encouraged to explicitly provide information about the time scale they are referring to when interpreting their results with respect to refugial isolation or range expansions (Qiu et al., 2011). Concerning the uncertainties about the glacial extent (see section “Quaternary Glaciations in the THR”), the available plant phylogeographic studies don’t seem support the existence of a large ice sheet covering most of the THR during the Last Glacial. Instead, populations of many species likely survived unfavorable conditions in refugia (see section “Main Phylogeographic Patterns of Seed Plant Species in the THR”).

Studies considering all the aspects mentioned in this review will provide valuable future insights into the phylogeography of the THR’s seed plants, for example, into trait relatedness to phylogeographic patterns, genetic discontinuities in relation to biogeographic barriers (e.g., Mekong-Salween Divide, compare Li et al., 2011; between Sino-Himalayan and Sino-Japanese Forest subkingdoms in the Sichuan Basin area, compare Qiu et al., 2011; glaciers and nunatak survival), the impact of geographic connectivity and separation of populations (e.g., also in relation to ancient drainage systems, compare Zhang et al., 2011; Yue et al., 2012; Chen et al., 2013), both in terms of frequency and duration through time, on population genetic patterns and processes, and many others.

## Author Contributions

The author confirms being the sole contributor of this work and has approved it for publication.

## Conflict of Interest Statement

The author declares that the research was conducted in the absence of any commercial or financial relationships that could be construed as a potential conflict of interest.
