# Supplementary material for: Mountains as Evolutionary Arenas: Patterns, Emerging Approaches, Paradigm Shifts, and Their Implications for Plant Phylogeographic Research in the Tibeto-Himalayan Region
Source: Front Plant Sci. 2019 Mar 18;10:195. doi: 10.3389/fpls.2019.00195 (PMC6431670; doi:10.3389/fpls.2019.00195)
Supplement: Supplementary file 1 [file Table_1.docx]

Supplementary Material

# Supplementary Table

**Table S1.** Phylogeographic patterns of seed plant species in the Tibeto-Himalayan region (THR), which comprises the Qinghai–Tibetan Plateau (QTP), the Himalayas, and the biodiversity hotspot known as the “Mountains of Southwest China” (Hengduan Mountains region): ‘‘contraction/recolonization”, ‘‘platform refugia/local expansion”, and ‘‘microrefugia”. Only studies of plant species with a current distribution including the QTP were considered, several of them additionally occurring in the adjacent Himalayas and/or the Hengduan Mountains region (i.e., a subset of the 90+ studies mentioned in the introduction). Species merely occurring in the mountain systems adjacent to the QTP (only Himalayas and/or Hengduan Mountains region) were not considered. List of references is not exclusive. Studies in English language published up to August 2018 were considered. Studies for each of the three main phylogeographic patterns are listed alphabetically according to species name. N: see note below table.

| Pattern/Species | Family | Reference(s) |
| --- | --- | --- |
| “contraction/recolonization” |  |  |
| *Allium cyathophorum, A. spicata* | Amaryllidaceae | Wang et al., 2015 |
| *Allium przewalskianum* | Amaryllidaceae | Wu et al., 2010 |
| *Angelica nitida* | Apiaceae | Zhang et al., 2013 |
| *Bupleurum smithii* | Apiaceae | Zhao et al., 2013 |
| *Gentiana lawrencei* var. *farreri* | Gentianaceae | Fu et al., 2018^N1^ |
| *Hippophaë neurocarpa* | Elaeagnaceae | Kou et al., 2014 |
| *Juniperus przewalskii* | Cupressaceae | Zhang et al., 2005* |
| *Koenigia islandica* | Polygonaceae | Long et al., 2014 |
| *Metagentiana striata* | Gentianaceae | Chen et al., 2008* |
| *Pedicularis longiflora* | Orobanchaceae | Yang et al., 2008* |
| *Picea crassifolia* | Pinaceae | Meng et al., 2007* |
| *Quercus aquifolioides* | Fagaceae | Du et al., 2017* |
| *Rhodiola kirilowii* | Crassulaceae | Zhang et al., 2014 |
| *Sibiraea angustata* | Rosaceae | Fu et al., 2016^N2^ |
|  |  |  |
| “platform refugia/local expansion” |  |  |
| *Aconitum gymnandrum* | Ranunculaceae | Wang et al., 2009a* |
| *Gentiana straminea* | Gentianaceae | Lu et al., 2015* |
| *Medicago ruthenica* | Fabaceae | Wu et al., 2016 |
| *Pomatosace filicula* | Primulaceae | Wang et al., 2014* |
| *Potentilla glabra* | Rosaceae | Wang et al., 2009b* |
| *Ranunculus bungei* (syn. *Batrachium bungei)* | Ranunculaceae | Wang et al., 2010*; Chen et al., 2014* |
| *Rhodiola alsia* | Crassulaceae | Gao et al., 2009*, 2012*^N3^ |
|  |  |  |
| “microrefugia” |  |  |
| *Anisodus tanguticus* | Solanaceae | Wan et al., 2016 |
| *Clintonia udensis* | Liliaceae | Wang et al., 2010 |
| *Hippophae tibetana* | Elaeagnaceae | Wang et al., 2010*; Jia et al. 2011* |
| *Juniperus tibetica* agg. | Cupressaceae | Opgenoorth et al., 2010* |
| *Orinus thoroldii* | Poaceae | Liu et al., 2015 |
| *Picea likiangensis*, *P. wilsonii*, *P. purpurea* | Pinaceae | Li et al., 2010b* |
| *Pinus densata* | Pinaceae | Wang et al., 2011 |
| *Potentilla fruticosa* | Rosaceae | Li et al., 2010a*; Shimono et al., 2010*; Sun et al. 2010* |
| *Primula tibetica* | Primulaceae | Ren et al., 2017 |
| *Rhodiola chrysanthemifolia* | Crassulaceae | Gao et al., 2016* |
| *Rhodiola sect. Trifida* | Crassulaceae | Li et al., 2018 |
| *Saxifraga sinomontana* | Saxifragaceae | Li et al., 2018* |
| *Spenceria ramalana* | Rosaceae | Yue et al., 2014 ^N4^ |
| *Spiraea alpina* | Rosaceae | Zhang et al., 2012 |
| *Taxus wallichiana* | Taxaceae | Gao et al., 2007*, Liu et al., 2013* |

N1: Fu et al.(2018) also additionally suggest the existence of one microrefugium on the QTP proper.

N2: Fu et al.(2016) also additionally suggest the existence of a microrefugium on the QTP proper.

N3: Gao et al. (2012) also reported an additional refugium in the Hengduan Mountains region.

N4: Potential refugia were located at the margin areas of the species´ current distribution range.

**References**

(Note: Only those references not yet included in the main manuscript text are given below; references included in the main text are marked by an asterisk “*” in the table above)

Fu, P. C., Gao, Q. B., Zhang, F. Q., Xing, R., Khan, G., Wang, J. L., et al. (2016). Responses of plants to changes in Qinghai–Tibetan Plateau and glaciations: evidence from phylogeography of a *Sibiraea* (Rosaceae) complex. *Biochem.* *Syst. Ecol.* 65, 72–82. doi: 10.1016/j.bse.2016.01.006

Fu, P.-C., Ya, H.-Y., Liu, Q.-W., Cai, H.-M., and Chen, S.-L. (2018). Out of refugia: population genetic structure and evolutionary history of the alpine medicinal plant *Gentiana lawrencei* var. *farreri* (Gentianaceae). *Front. Genet.* doi.org/10.3389/fgene.2018.00564

Kou, Y.X., Wu, Y.X., Jia, D.R., Li, Z.H., and Wang, Y.J. (2014). Range expansion, genetic differentiation, and phenotypic adaption of *Hippophaë neurocarpa* (Elaeagnaceae) on the Qinghai-Tibet Plateau. *J Syst. Evol.* 52, 303–312. doi: 10.1111/jse.12063

Li, Y. C., Zhong, D. L., Rao, G. Y., Wen, J., Ren, Y., and Zhang, J. Q. (2018). Gone with the trees: phylogeography of *Rhodiola* sect. *Trifida* (Crassulaceae) reveals multiple refugia on the Qinghai-Tibetan Plateau. *Mol. Phylogenet. Evol.* 121, 110–120. doi: 10.1016/j.ympev.2018.01.001

Liu, Y., Su, X., He, Y., Han, L., Huang, Y., and Wang, Z. (2015). Evolutionary history of *Orinus thoroldii* (Poaceae), endemic to the western Qinghai-Tibetan Plateau in China. *Biochem. Syst. Ecol.* 59, 159–167. doi: 10.1016/j.bse.2015.01.014

Long, C., Min, Y., Zhao, X., Yany, C., Sun, H., Lü, H., Tang, L., and Zhou, Z. (2014). Origin area and migration route: Chloroplast DNA diversity in the arctic-alpine plant *Koenigia islandica*. *Science China: Earth Sciences*, 57, 1760–1770. doi: 10.1007/s11430-014-4819-2

Ren, G., Mateo, G., Liu, J., Suchan, T., Alvarez, N., Guisan, A., Conti, E., and Salamin, N. (2017). Genetic consequences of Quaternary climatic oscillations in the Himalayas: *Primula tibetica* as a case study based on restriction site‐associated DNA sequencing. *New Phytol.* *213*, 1500–1512.

Wan, D.S., Feng, J.J., Jiang, D.C., Mao, K.S., Duan, Y.W., Miehe, G., and Opgenoorth, L. (2016). The Quaternary evolutionary history, potential distribution dynamics, and conservation implications for a Qinghai-Tibet Plateau endemic herbaceous perennial, *Anisodus tanguticus* (Solanaceae). *Ecol. Evol.* 6, 1977–1995. doi: 10.1002/ece3.2019

Wang, Y. L., Li, X., Guo, J., Li, S. F., and Zhao, G. F. (2010). Chloroplast DNA phylogeography of *Clintonia udensis* Trautv. & Mey. (Liliaceae) in East Asia. *Molec. Phylogen. Evol.* 55, 721–732.

Wang, B., Mao, J.F., Gao, J., Zhao, W., and Wang, X.R. (2011). Colonization of the Tibetan Plateau by the homoploid hybrid pine *Pinus densata*. *Molec. Ecol.* 20, 3796–3811. doi: 10.1111/j.1365-294X.2011.05157.x. Epub 2011 Jun 20.

Wu, X., Liu, D., Gulzar, K., Shen, Y., and Wang, H. (2016). Population genetic structure and demographic history of *Medicago ruthenica* (Fabaceae) on the Qinghai-Tibetan Plateau based on nuclear ITS and chloroplast markers. *Biochem. Syst. Ecol.* 69, 204–212. doi: 10.1016/j.bse.2016.10.005

Zhang, F.Q., Gao, Q.B., Zhang, D.J., Duan, Y.Z., Li, Y.H., Fu, P.C., Xing, R., Gulzar, K., and Chen, S.L. (2012). Phylogeography of *Spiraea alpina* (Rosaceae) in the Qinghai-Tibetan Plateau inferred from chloroplast DNA sequence variations. *J. Syst. Evol.* 50, 276–283. doi: 10.1111/j.1759-6831.2012.00194.x

Zhang, J., Meng, S., and Rao, G. (2014). Phylogeography of *Rhodiola kirilowii* (Crassulaceae): A story of Miocene divergence and Quaternary expansion. *PLoS ONE*, 9, e112923. doi: 10.1371/journal.pone.0112923
