# Supplementary material for: Mountains as Evolutionary Arenas: Patterns, Emerging Approaches, Paradigm Shifts, and Their Implications for Plant Phylogeographic Research in the Tibeto-Himalayan Region
Source: Front Plant Sci. 2019 Mar 18;10:195. doi: 10.3389/fpls.2019.00195 (PMC6431670; doi:10.3389/fpls.2019.00195)
Supplement: Supplementary file 2 [file Data_Sheet_1.docx]

Supplementary Material

# Supplementary Figure

**Supplementary Figure S1.** Selected reconstructions for the maximum extent of glaciation during the Last Glacial across Tibet and the bordering mountains of the Tibeto-Himalayan region (THR). “Light brown, relief over 4000 m above sea level; dark blue-gray, areas considered glaciated. A) Klute’s (1930) reconstruction based on a temperature depression of ~4 °C, with a shift of climatic zones to the south and an intensification of atmospheric circulation such that precipitation increased towards the dry areas of central Asia. B) Frenzel’s (1960) reconstruction based on the detailed work of Von Wissmann (1959), who evaluated the observations of the earliest explorers. C) Kuhle’s (1985) reconstruction based on field observations and extrapolation of large equilibrium-line altitude depressions (>1000 m) from the margins of Tibet into the interior regions. D) Reconstruction of Shi (1992) and Li et al. (1991) based on detailed field mapping of glacial and associated landforms and sediments.” Reprinted from Quaternary Science Reviews, 88, Owen, L.A., and Dortch, J.M., Nature and timing of Quaternary glaciation in the Himalayan-Tibetan orogen, pp. 14-54, 2014, with permission from Elsevier. © 2013 Elsevier Ltd. https://www.sciencedirect.com/science/article/abs/pii/S0277379113004599.

**References**

Frenzel, B. (1960). Die Vegetations- und Landschaftszonen Nord-Eurasiens während der letzten Eiszeit und während der postglazialen Wärmezeit. *Akademie der Wissenschaften und der Literatur Mainz, Abhandlungen der Mathematisch-Naturwissenschaftlichen Klasse* 13, 937–1099.

Klute, F. (1930). Verschiebung der Klimagebiete der letzten Eiszeit. *Petermanns Mitteilungen, Ergänzungsheft* 209, 166–182.

Kuhle, M. (1985). Ein subtropisches Inlandeis als Eiszeitauslöser. Südtibet- und Mt. Everest-Expedition 1984. *Georgia Augusta, Nachrichten Universität Göttingen* 42, 35–51.

Li, B., Li, J., Cui, Z., Zheng, B., Zhang, Q., Wang, F., Zhou, S., Shi, Z., Jiao, K., Kang, J. (1991). Quaternary glacial distribution map of Qinghai-Xizang (Tibet) Plateau. Beijing, China: Science Press.

Shi, Y. (1992). Glaciers and glacial geomorphology in China. *Zeitschrift für Geomorphologie, Supplement-Band* 86, 51–63.

Von Wissmann, H. (1959). Die heutige Vergletscherung und Schneegrenze in Hochasien. *Akademie der Wissenschaften und der Literatur Mainz, Abhandlungen der Mathematisch-Naturwissenschaftlichen Klasse* 14, 1101–1407.
